# Supplementary figures and images for: TWIST1 Drives Cytotoxic CD8+ T-Cell Exhaustion through Transcriptional Activation of CD274 (PD-L1) Expression in Breast Cancer Cells
Source: Cancers (Basel). 2024 May 22;16(11):1973. doi: 10.3390/cancers16111973 (PMC11171171; doi:10.3390/cancers16111973)

Figure 1E

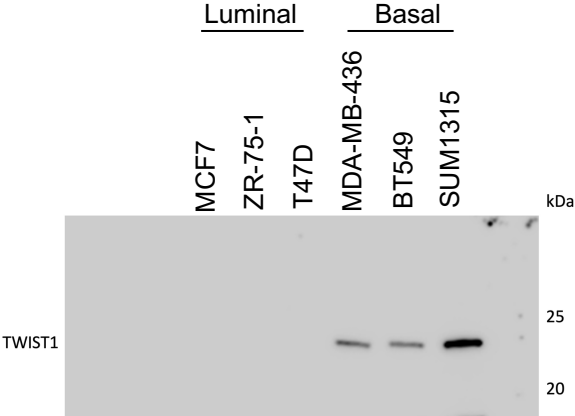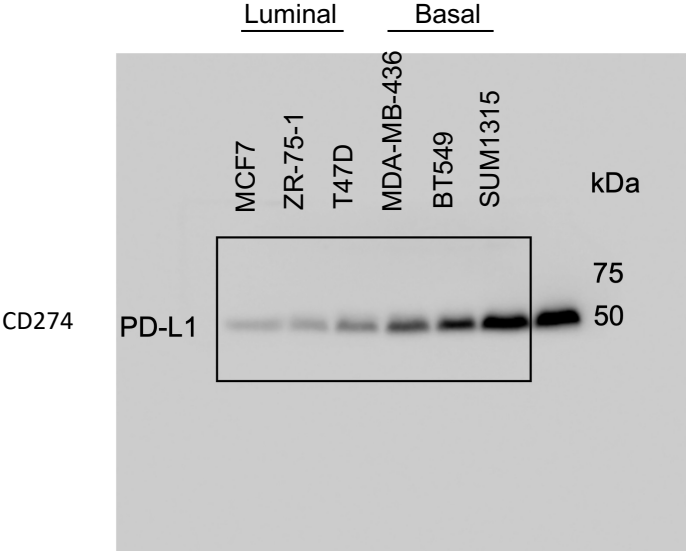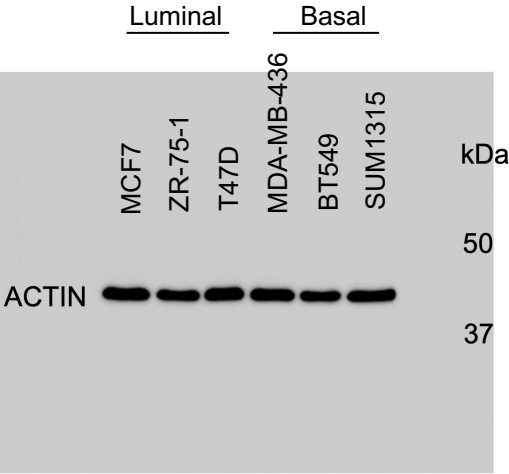

**Figure 2B**

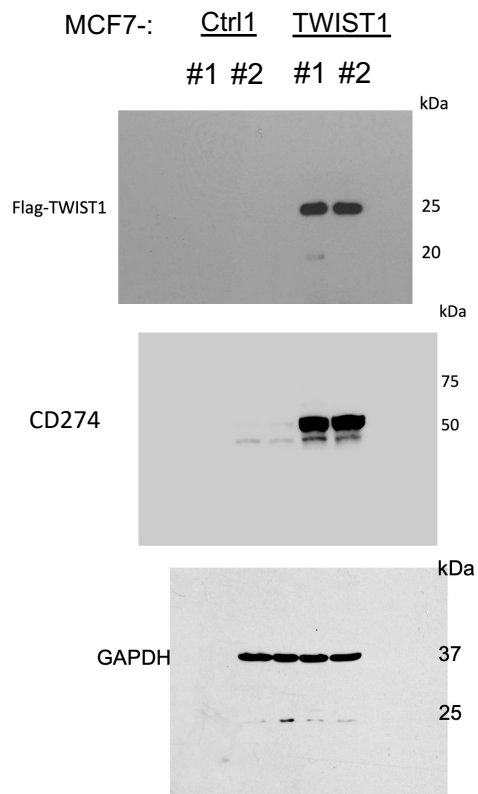

**Figure 2D**

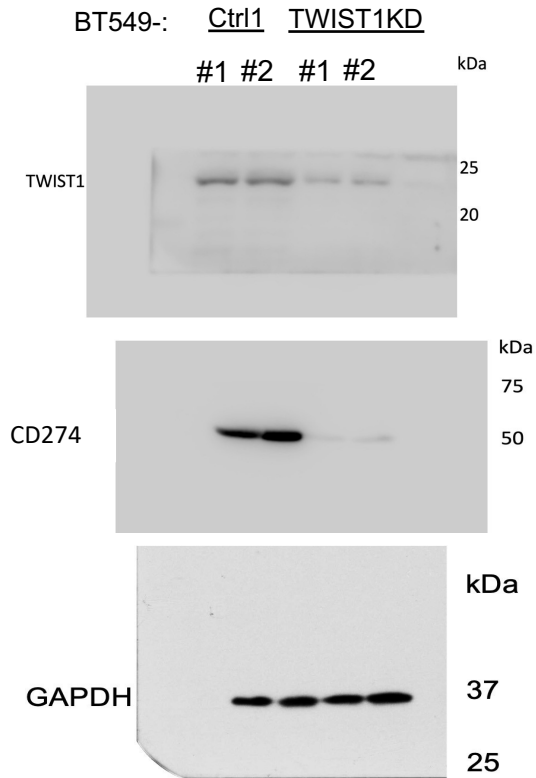

Figure 4B

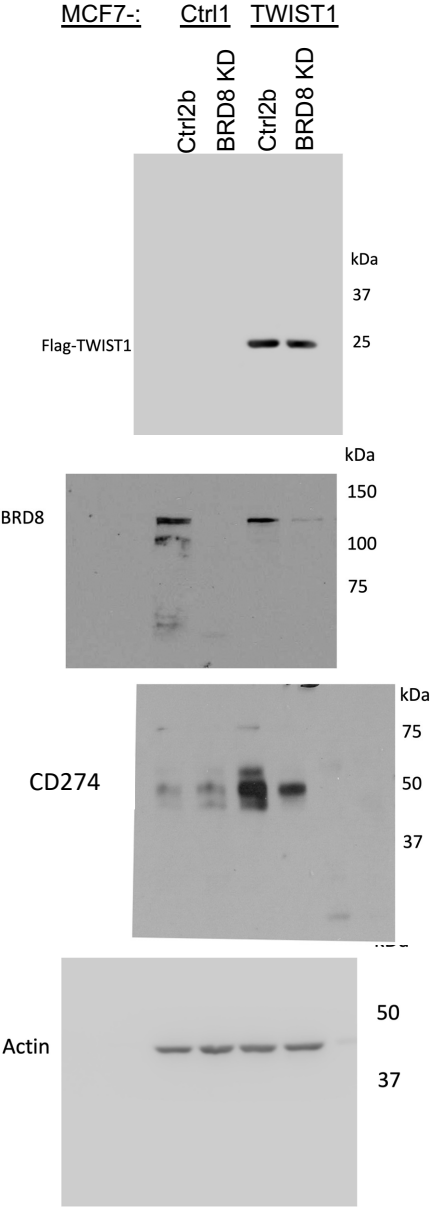

Figure 4D

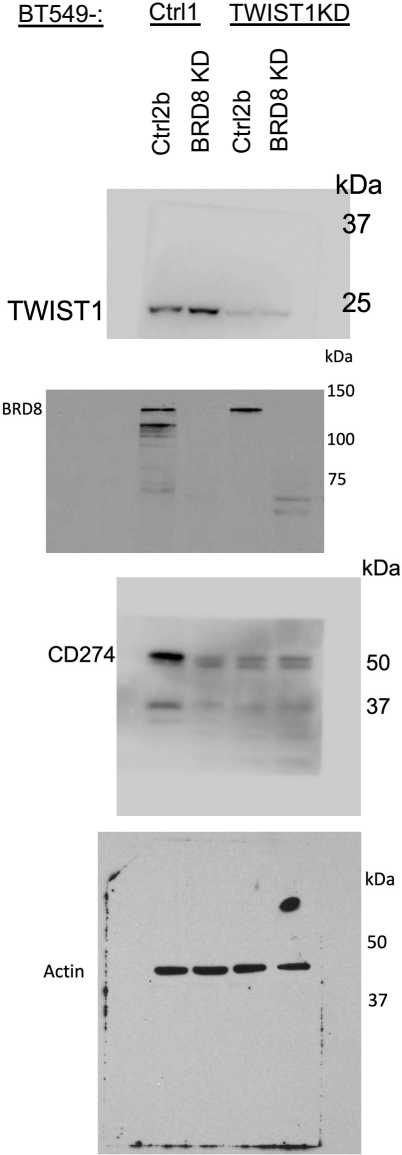

Figure 5

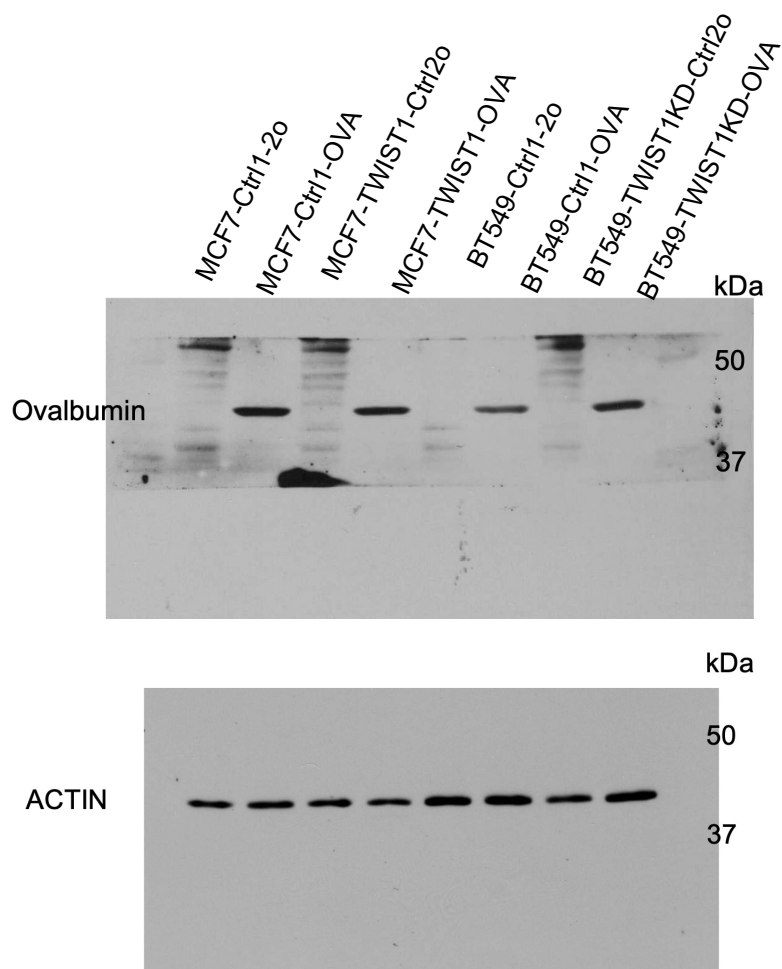

Supplement: Supplementary file 1 [file cancers-16-01973-s001.zip › cancers-2986531-supplementary.pdf]
